# A retrospective evaluation of short-term results from colonic stenting as a bridge to elective surgery versus emergency surgery for malignant colonic obstruction

**DOI:** 10.1038/s41598-023-28685-y

**Published:** 2023-01-28

**Authors:** Chongjing Mu, Lei Chen

**Affiliations:** 1 The Affiliated Suzhou Hospital of Nanjing Medical University, 16 Baita West Road, Suzhou, 215000 People’s Republic of China; 2 Gusu School, Nanjing Medical University, 458 Shizi Road, Suzhou, 215000 People’s Republic of China

**Keywords:** Gastroenterology, Oncology

## Abstract

The efficacy and safety of self-expanding metallic stent (SEMS) placement as a bridge to elective surgery versus emergency surgery to treat malignant colonic obstruction is debated. This study aimed to evaluate the outcomes of patients with malignant colonic obstruction treated using different procedure. Subjects admitted to the authors’ department with colonic obstruction (n = 87) were studied. They underwent colonic stenting as a bridge to elective surgery (SEMS group: n = 14) or emergency surgery (ES group: n = 22).Their demographic characteristics, stoma rate, laparoscopy rate and postoperative complications were analyzed, and the potential risk factors of postoperative complications and the optimal time interval from SEMS implantation to elective surgery were explored. The stoma rate was 15.4% in the SEMS group versus 60.0% in the ES group (P = 0.015), and the postoperative complication rate was 7.7% in the SEMS group versus 40.0% in the ES group (P = 0.042). The proportion of patients undergoing laparoscopy in SEMS group was significantly higher than that in ES group (69.2% vs. 15.0%; P = 0.003).The effect of ASA grade on postoperative complications was statistically significant (OR = 24.565; P = 0.008). The Receiving operating characteristic (ROC) curve could not determine the optimal time interval between SEMS implantation and elective surgery (AUC = 0.466). SEMS implantation has the advantages of lower temporary stoma rate, less postoperative complications and higher laparoscopy rate compared with ES in the treatment of left malignant intestinal obstruction. ASA grade is a risk factor for postoperative complications. However, larger sample size prospective randomized controlled trials (RCT) are still needed to confirm long-term oncological outcomes.

## Introduction

Colon cancer is the most common type of malignancy in the world, particularly in economically developed countries, and the incidence of colon cancer remains high in China as well^1^. Colon cancer patients typically present with altered fecal characteristics and bowel habits, as well as abdominal pain and distension. There is a thin lumen on the left colon and hard feces, and the histological type is infiltrative, which may result in annular stenosis, so approximately 10–15% of patients with colon cancer initially manifest complete or incomplete intestinal obstruction^2,3^, of which 75% of patients develop ileus in the left colon. Patients who suffer obstructions typically undergo surgical emergency surgery (ES) in order to eliminate the obstruction. There has been study showing a mortality rate of 15–20% in ES, and complications range from 45 to 81%.^4^. Consequently, self-expanding metal stent (SEMS) have been increasingly used for preoperative transition since 1991 and have been shown to be a safe and effective treatment option for obstructive colon cancer. Because of more appropriate preoperative preparation, tumor resection after stenting results in a lower complication rate and mortality rate compared to ES, which are only 0.9–6%.^4^. However, three randomized controlled trials (RCT) investigating the advantages of SEMS over ES in recent years were prematurely terminated. This is mainly because of the high rate of perforation and anastomotic leak in the SEMS group, the technical success rate of SEMS placement is low without difference in stoma rate^5–7^. Currently, the use of colonic stents in malignant intestinal obstruction patients remains controversial^8,9^. This study collected data of patients with acute intestinal obstruction who received different treatments in our hospital from January 2014 to October 2021, and aimed to analyze the short-term outcomes of two methods, ES and SEMS placement, for left colon cancer with obstruction.

## Materials and methods

### Patient identification and enrollment

This study was approved by the ethics committee of The Affiliated Suzhou Hospital of Nanjing Medical University. In this study, we reviewed patient data from left colon cancer patients with obstruction at our hospital between January 2014 and October 2021 and divided them into two groups according to whether they received stent placement (SEMS group) or emergency surgery (ES group). Inclusion criteria: (1) patients who had symptoms such as abdominal discomfort or distention prior to surgery, or imaging shows such as bowel distension or air-fluid level; (2) postoperative diagnosed with colon cancer by histopathology; (3) the primary tumor was located between the splenic flexure and the rectosigmoid junction. Exclusion criteria: (1) multisite obstruction or obstruction of the small bowel, or large bowel perforation, peritonitis, intestinal adhesion; (2) surgery was not performed after SEMS placement on the tumor; (3) patients who has to undergo emergency surgery because of a difficult stent placement or complications related to the stent.

### Methods

#### SEMS placement

All patients were evaluated by a routine physical examination during admission to determine their general health status. The length of obstruction was estimated based on the extent and location of the tumor assessed by endoscopy and computed tomography (CT). Propofol infusion was used under anesthetist supervision during the procedure. A nitinol uncovered stent was inserted through the working channel of the endoscope. The procedure was stopped in cases of significantly varied patient vital signs, uncontrolled pain, or significant abdominal distension, or if the relative position between the colonoscope and the visible part of the stricture was not stable. The SEMS placement procedure was performed by 2 experienced interventional physicians. If the patient has severe complications (includes perforation, bleeding, stent displacement, reobstruction, colonic obstruction symptoms not relieved) after stent placement that do not respond to conservative treatment, the patient is transitioned to emergency surgery. The decision to refer a patient for emergency surgery or for SEMS placement with a later elective operation was made by the senior on-call surgeon. The type of operation and the technique to be used were determined by the surgeon according to the location of the primary disease and the intraoperative conditions. The interval from SEMS placement to elective surgery also was chosen by the attending surgeon based on clinical condition and bowel function.

#### ES

Patients in the ES group were routinely prepared preoperatively and underwent emergency surgery in a timely manner. As noted, the type of operation to be used were determined by the surgeon and endoscopist according to the intraoperative conditions. Colostomy is considered an optional surgical procedure.

### Outcome measures

Data on demographics, comorbidities, tumor location, stent related complications, surgical approach, whether or not a stoma was created, operative time, number of lymph nodes dissected, postoperative hospital stay, histopathological findings, and postoperative complications were collected. The success rates of stent placement, including technical success and clinical success, were analyzed. Technical success was defined as whether stent placement was successful. Clinical success was defined as colonic decompression and relief of obstructive symptoms within 24 h for those patients who had SEMS placed, and no stent related complications have occurred until the time of elective surgery.

### Statistics

All data were processed and analyzed using Statistical Package for Social Sciences version 26.0 for Windows (SPSS Inc., Chicago, IL, USA). Quantitative data that met normal distribution were reported as mean ± standard deviation (x ± s), and the comparison of means between two samples was performed by t-test. Qualitative data were reported as frequency (n) and percentage (%), and chi-square test or Fisher's exact test was performed; multiple logistic regression models were used to evaluate risk factors associated with surgical complications. The Receiving operating characteristic (ROC) curve analysis was used to determine the optimal time interval from SEMS placement to elective surgery. P < 0.05 was considered statistically significant.

### Ethics approval and consent to participate

This research study was conducted retrospectively from data obtained for clinical purposes. This study was approved by the Ethics Committee of The Affiliated Suzhou Hospital of Nanjing Medical University. We obtained informed consent for all patients. All methods were performed in accordance with the relevant guidelines and regulations.

## Outcome

**Figure 1:**
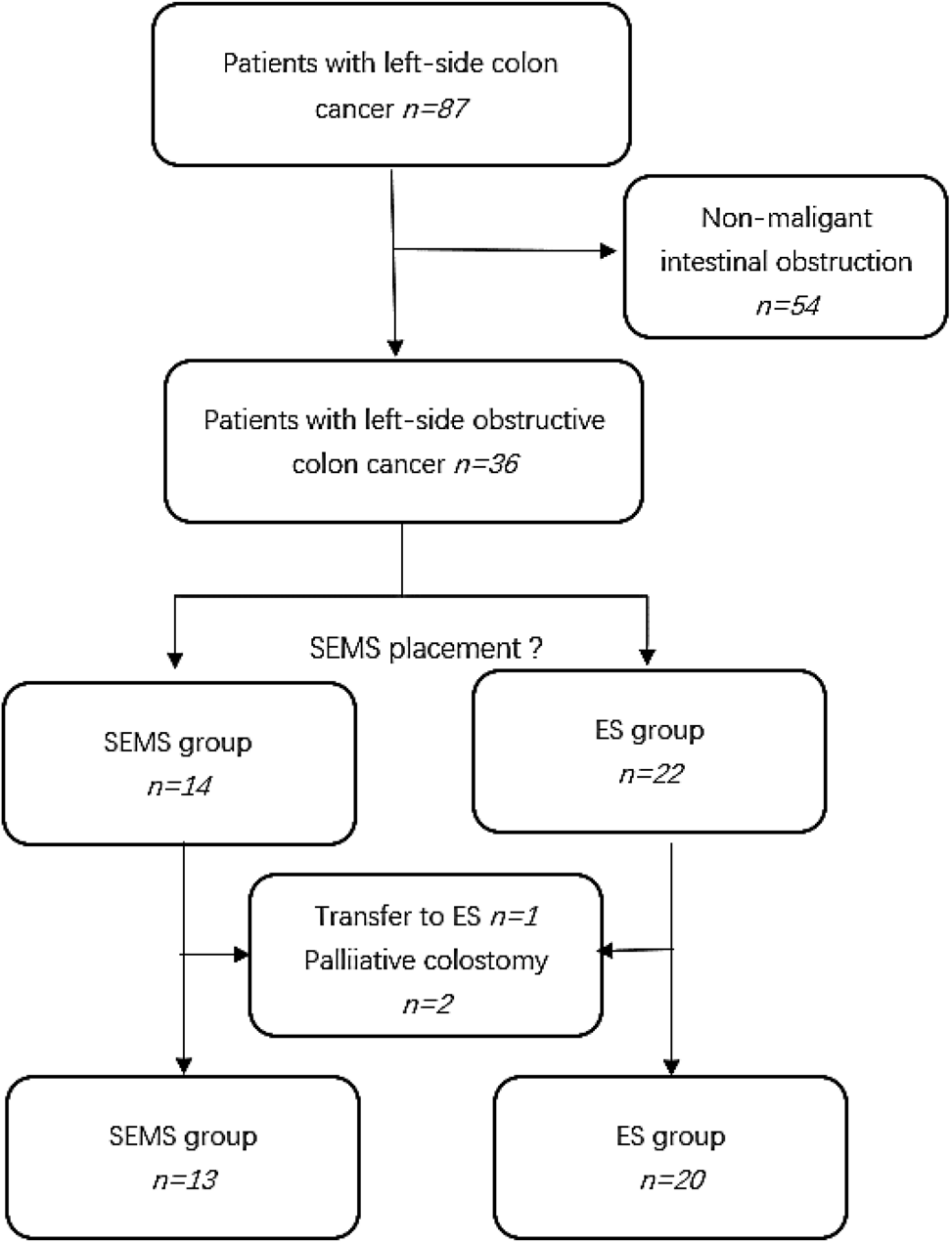
Patient analysis flowchart.

A total of 87 patients with left colon cancer were collected, including 54 patients with intestinal obstruction due to non malignancy, 22 patients who underwent ES, and 14 patients who underwent SEMS. Three were excluded because of the obstruction was not relieved (n = 1) and resection was not performed in the advanced stage of the tumor (n = 2) according to the inclusion and exclusion criteria. Finally, 13 were included in the SEMS group and 20 in the ES group. A flowchart of patient allocation is shown in Fig. 1.

There were no statistical differences in gender, mean age, body mass index (BMI), and tumor location between the two groups. There was no significant difference in pathological stage between the two groups (P = 0.550) (Table 1). The technical success rate of stenting in the SEMS group was 100% (14/14), with 1 patient whose symptoms of intestinal obstruction were not relieved and who was transferred to the ES. The clinical remission rate of 93% (13/14). The mean time interval between SEMS placement and elective surgery was 18 days (11–41 days), with two patients having significantly longer wait times due to preoperative chemotherapy. There were no statistically significant differences in operative time, number of lymph nodes harvested and postoperative hospital stay. 12 temporary or permanent stomas in the ES group, a lower stoma rate in the SEMS group (15.4% vs. 60.0%; P = 0.015) and a significantly higher proportion of patients undergoing laparoscopy in the SEMS group (69.2% vs. 15.0%; P = 0.003) (Table 2). The incidence of postoperative complications was higher in the ES group than in the SEMS group (7.7% vs. 40.0%; P = 0.042) (Table 3).

**Table 1:** Patient characteristics of patient in SEMS group vs. ES group.

	SEMS (n = 13)	ES (n = 20)	p value
Age (years, mean ± SD)	68.69 ± 13.55	64.70 ± 12.35	0.389*
Gender (male/female)	10/3	15/5	
BMI (kg/m^2^, mean ± SD)	21.25 ± 3.60	23.65 ± 3.08	0.05*
Diabetes (%)	1 (7.7)	6 (30.0)	0.20^#^
Hypertension (%)	6 (46.2)	5 (25.0)	0.27^#^
Site of tumor (%)			
Splenic flexure of colon	0 (0)	3 (15.0)	0.399^#^
Descending colon	8 (61.5)	11 (55.0)	
Sigmoid	3 (23.1)	5 (25.0)	
Rectosigmoid	2 (15.4)	1 (5.0)	
ASA classification (%)			
1	5 (38.5)	9 (45.0)	0.881^#^
2	7 (53.8)	9 (45.0)	
3	1 (7.7)	2 (10.0)	
Stage (%)			
II	4 (30.8)	7 (35.0)	0.550^#^
III	5 (38.5)	10 (50.0)	
IV	4 (30.8)	3 (15.0)	

*t test.

^#^Chi-square test or fisher's exact test.

**Table 2:** Operative outcomes.

	SEMS (n = 13)	ES (n = 20)	p value
Operative time (min, mean ± SD)	146.92 ± 48.76	148.25 ± 59.92	0.947*
Lymph nodes (mean ± SD)	16.30 ± 6.96	13.50 ± 4.31	0.161*
Postoperative hospital stay (day)	14.15 ± 4.24	14.25 ± 5.61	0.958*
Perineural invasion (%)	4 (30.8)	7 (35.0)	1.00^#^
Approach (%)			
Laparoscopic	9 (69.2)	3 (15.0)	0.003^#^
Open	4 (30.8)	17 (85.0)	
Stoma (%)			
Yes	2 (15.4)	12 (60.0)	0.015^#^
No	11 (84.6)	8 (40.0)	

*t test.

^#^Chi-square test or fisher's exact test.

**Table 3:** Postoperative complications.

	STBS (n = 13)	ES (n = 20)	p value
Overall complication (%)	1 (7.7)	8 (40.0)	0.042^#^
Wound infection	0 (0)	3 (15.0)	
Pneumonia	1 (7.7)	5 (25.0)	
Anastomotic leakage	0 (0)	2 (10.0)	

*t test.

^#^Chi-square test or fisher's exact test.

Age, American Society of Anesthesiologists (ASA) grade, and SEMS placement were included to construct multivariable logistic regression equations. It was found that there was no statistical significance regarding the effect of age or SEMS placement on postoperative complications (OR = 1.0, 95% CI 0.9–1.1, P = 0.6; OR = 0.1, 95% CI 0.01–1.5, P = 0.1); the effect of ASA grade on postoperative complications was statistically significant (OR = 24.6, 95% CI 2.3–263.3, P = 0.008) (Table 4).

**Table 4:** Multivariable logistic regression analysis.

Covariates	Odds ratio (95% confidence interval)	p value
Age	1.023 (0.942–1.110)	0.589
SEMS implantation	0.130 (0.011–1.504)	0.102
ASA classification	24.565 (2.292–263.259)	0.008

**Figure 2:**
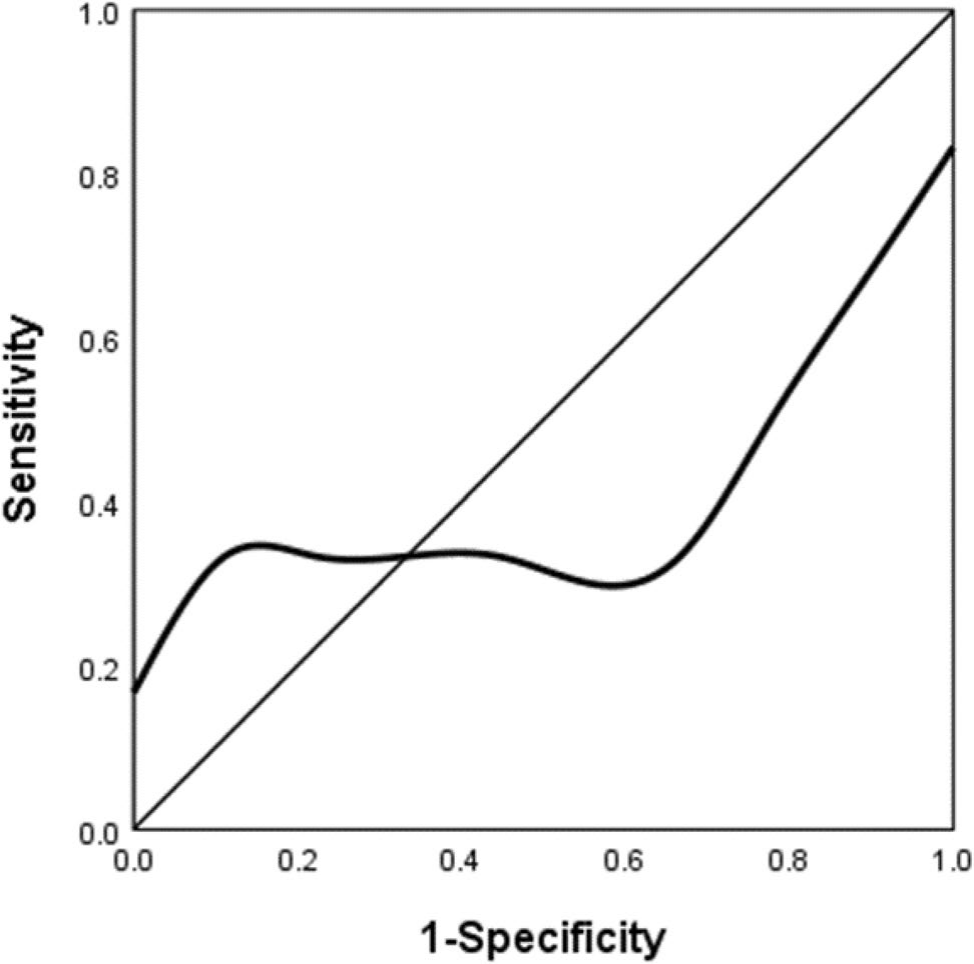
Receiving operating characteristic (ROC) curve showing the trade-off between sensitivity and specificity by considering from interval between SEMS placement and surgery to the occurrence of postoperative complications (n = 12).

The ROC curve could not identify the optimal time interval between SEMS placement and elective surgery (AUC = 0.466) (Fig. 2).

## Discussions

According to previous studies, the success rate of ES rather than colostomy is about 28–41%^10,11^. It is usually accompanied by a variety of complications and higher mortality and stoma rate, which result in the terrible quality-of-life. SEMS placement allows doctors ample time to evaluate a patient's tumor staging and avoid unnecessary surgery. It also can reduce perioperative complications and stoma. This provides the opportunity to doctors a variety of treatments, such as preoperative chemotherapy and laparoscopy for malignant colon obstruction. Contrasting previous RCT^5–7^, the technical success rate was 100% and the clinical success rate was 93% in our study. This shows that SEMS placement needs to be done by specialized endoscopist, who need to master more techniques in stent placement. It has been found that age and site of obstruction were significantly associated with intestinal perforation^12^. Particularly in the splenic flexure of the colon, the angle of curvature of the lumen is further increased as intestinal dilatation, making it more difficult and leading to perforation at the time of stent placement. There was only 1 case in this study in which the symptoms of colonic obstruction did not relieve after SEMS placement, and no other stent related complications occurred.

Cirocchi et al. reported that there was no advantage of SEMS placement for malignant obstruction of left-sied colon in terms of complications and postoperative mortality^13^, but it can increase the primary anastomosis rate and reduce the stoma. This is critical because it can affect the quality of life of the patient and thus have an impact on the overall health of the patient^14^. There were no postoperative deaths in this study, which may be related to the small sample size. Consistent with the results of some RCT^7,15–17^, SEMS group was significantly better than ES group in postoperative complications, primary anastomosis rate and stoma rate, which may be related to the fact that it can improve patients' clinical condition and bowel function before elective surgery.

Laparoscopy is affected by dilation of the small intestine and the proximal colon, making it difficult to perform it for obstructive colon cancer, although it carries the advantages of shorter hospital stays, faster postoperative recovery, and easier control of the immune and inflammatory responses^18^. SEMS placement can save enough time for bowel preparation and recovery of clinical condition to allow for laparoscopy. In the present study, we found that laparoscopy was performed more frequently in the SEMS group (69% vs. 15%; P = 0.003). This is comparable to research conducted by Law^19^ and Seung et al.^20^, the latter chose laparoscopy technology after using SEMS.

Many studies have shown that SEMS placement does not decrease survival^7,15–17,21^. However, Sabbagh et al.^22^ noted that the SEMS group had a significantly worse overall survival than the ES group (25% vs. 62%, P = 0.0003). Sloothaak et al.^23^ explained that SEMS placement may increase the risk of recurrence because there is a higher recurrence rate in patients with perforations. Stent-related complications are closely related to stent implantation technology, so the success rate of stent implantation is the first problem to be solved. It has also been found that SEMS placement can change perineural invasion and lymphatic invasion, and negatively affect the long-term prognosis of patients^24^. The reason may be that the compression of the tumor after the placement of SEMS and the creation of silent perforation of the intestine^5^ promote the progression and metastasis of the tumor. Many studies suggest that SEMS implantation should only be performed in centre with experienced endoscopists because of the uncertainty of the impact of SEMS implantation on tumor outcome. Therefore, the impact of SEMS placement on tumor characteristics and patients' long-term outcomes still needs further investigation.

Postoperative complications are important factors affecting surgical outcomes and patients' quality of life, therefore, it is necessary to minimize postoperative complications as much as possible. Age, ASA grade and SEMS were included to construct a multivariate logistic regression equation in this study. We found that ASA grade was a risk factor for postoperative complications. So, colonic stenting allows for a more thorough and detailed preoperative evaluation to lower ASA grade and enhance anesthesia tolerance. The difference in clinical efficacy between the SEMS group and the ES group may have been achieved by lowering the ASA grade. Comply with the guidelines of the European Society for Gastrointestinal Endoscopy (ESGE): SEMS apply to patients with ASA grade ≥ III/aged > 70 years^25^. However, there was no statistical difference in the age and placement of SEMS in this study. The reason may be that small sample size limits statistical performance.

The optimal time interval from SEMS placement to elective surgery remains uncertain. We hypothesized that the clinical benefit of an optimal interval manifests in postoperative complications, then a time point or period needs to be determined such that postoperative complications are minimal. The results showed that different time intervals were not associated with postoperative complications, which was consistent with the results of a previous study^26^. The relationship between time interval and overall survival rate and recurrence rate can be further considered to find the clinical significance of the best time interval from the long-term results.

This study is limited by a small sample size retrospective study, and other statistical differences between the two groups may be omitted. This study is a non randomized trial, which may have selection bias, because some patients with more serious condition, more significant intestinal dilatation and worse general condition were selected into the ES group, resulting in worse results in the ES group.

## Conclusions

SEMS implantation has the advantages of lower temporary stoma rate, less postoperative complications and higher laparoscopy rate compared with ES in the treatment of left malignant intestinal obstruction. ASA grade is a risk factor for postoperative complications. However, larger sample size prospective RCT are still needed to confirm long-term oncological outcomes.

## Data Availability

The datasets generated and/or analysed during the current study are not publicly available due the patient privacy agreement and further research but are available from the corresponding author on reasonable request.
